# A Finite Element Approximation for Nematic Liquid Crystal Flow with Stretching Effect Based on Nonincremental Pressure-Correction Method

**DOI:** 10.3390/e24121844

**Published:** 2022-12-18

**Authors:** Zhaoxia Meng, Meng Liu, Hongen Jia

**Affiliations:** 1 Department of Energy and Power Engineering, Shanxi Energy Institute, Taiyuan 030024, China; 2 School of Mathematics, Taiyuan University of Technology, Taiyuan 030024, China

**Keywords:** nematic liquid crystal, finite elements, nonincremental pressure-correction projection method, decoupled numerical scheme, 35Q35, 65M12, 76A15

## Abstract

In this paper, a new decoupling method is proposed to solve a nematic liquid crystal flow with stretching effect. In the finite element discrete framework, the director vector is calculated by introducing a new auxiliary variable w, and the velocity vector and scalar pressure are decoupled by a nonincremental pressure-correction projection method. Then, the energy dissipation law and unconditional energy stability of the resulting system are given. Finally, some numerical examples are given to verify the effects of various parameters on the singularity annihilation, stability and accuracy in space and time.

## 1. Introduction

Liquid crystal is a kind of material with excellent properties, which has been widely used in many new advanced technical fields. For example, display devices of the electronic industry and skin cancer examination in medicine. The defects, phase transition phenomenon, molecular distribution regularity, and dynamic behavior observed in liquid crystal are related to the quality of liquid crystal equipment, which are very important issues in liquid crystal technology and have attracted extensive attention of a large number of engineers and scientists.

Generally, according to the formation conditions of liquid crystals, they can be divided into thermotropic and lyotropic. The thermotropic liquid crystal is subdivided into smectic, nematic, and cholesteric based on symmetry. Among them, the nematic liquid crystal is widely used at present. Its molecules are rod-shaped. The molecular long axes are parallel to each other but not arranged in layers. They can slide up and down, to the left and right sides, front and back, or only be parallel or nearly parallel to each other in the molecular long-axis direction; the short-range interaction between the molecules is weak. The arrangement and movement of nematic liquid crystal molecules are relatively free, and they are quite sensitive to external forces. Nematic liquid crystal is currently the main material for making liquid crystal display devices.

From a statistical average point of view, the rods locally tend to be ordered, which can be described by a unit vector that represents the average direction, that is, the director vector d(x). The local directivity of liquid crystal material is easily changed by the external influences, such as the molecular orientation at the interface of different materials, electric field and magnetic field, etc., which leads to a change in material properties. With the change in molecular local direction, due to the discontinuity of the molecular local direction, defects (or singularities) often appear in the material.

Physicists and mathematicians have established various mathematical models for the study of liquid crystal. In the 1960s, Ericksen [1] and Leslie [2] proposed the hydrodynamics theory of liquid crystal. The Ericksen–Leslie nematic liquid crystal model is derived from a macroscopic point of view and involves many coupling terms between two vector domains. Because the whole system is too complicated, most of the research works are based on the simplification and approximation of the model.

When the fluid is incompressible, a simplified Ericksen–Leslie model is obtained [3]. It is composed of Navier–Stokes equations coupled with anisotropic elastic stress tensor and a convective harmonic mapping heat flow equation mapped onto the sphere. The model contains linear differential constraints and nonlinear algebraic constraints. The nonlinear terms in the model bring great difficulties to the theoretical analysis and the design of numerical examples. Especially, the restriction on director vector ∣d∣=1 must be satisfied everywhere. It is very difficult to satisfy the constraint condition almost everywhere in numerical simulation. Therefore, in order to relax the constraint condition ∣d∣=1, the penalty method and the saddle point method are proposed.

Lin-Lin proposed the penalized Ericksen–Leslie model. In the literature [4], the Galerkin method was used by them to prove the local existence of the classical solution and the global existence of the weak solution for the Dirichlet problem of the two-dimensional and three-dimensional penalized Ericksen–Leslie model, and the energy estimate was also given. For the numerical solution of the penalized Ericksen–Leslie equation, ref. [5] proposed a semi-implicit, first-order linear scheme, in which the function f(d) was fully explicitly processed, and the scheme was conditionally stable. In [6], a nonincremental pressure-correction projection scheme [7,8] was used, a pressure stabilization term [9] was added at the same time, and finally a linear and decoupled scheme was obtained. For the penalized Ericksen–Leslie equation with stretching effect, ref. [10] proposed a corrected modified midpoint scheme by using the finite difference method to study the formation of defects at the interface of liquid crystal. Ref. [11] used finite element method to obtain spatial discretization and the time-splitting method based on a nonincremental velocity correction projection scheme [8] to decouple variables. The literature in [12] used a spectral method to study this kind of problem. At the same time, there are many papers introducing different auxiliary variables ω to analyze the model [13,14,15]. Ref. [13] presents a conditionally stable fully discrete scheme when the time step satisfies certain constraints. On this basis, the paper by [14] introduces an auxiliary variable different from [13], giving a semi-implicit Euler time discrete scheme explicitly dealing with the Ginzburg–Landau penalized function, obtaining a coupled, linear, and unconditionally stable scheme.

The saddle point method [16], through introducing the Lagrange multiplier q to the equation of director vector d, can exert spherical constraint ∣d∣=1. Based on this idea, the penalty Ericksen–Leslie model can be unified. The authors give two unconditionally stable, conserved implicit schemes and a linear semi-implicit unconditionally stable scheme, semi-implicit with respect to the nonlinear term.

Compared with some existing works, a lot of works are devoted to study the nematic liquid crystal without stretching effect, which cannot be widely used in deformable devices. The research on the liquid crystal materials with stretching effect is of great practical significance, which can be widely used in deformable devices. However, there are few studies on this model in practice.

In this paper, we turned our attention to the Ericksen–Leslie model with stretching effect. First, we use the projection scheme of nonincremental pressure correction [8] to decouple the variables. We also give a proof of the energy stability of our decoupling system and discuss the annihilation of singularities. More precisely, we give details to take care of the relation among the stabilization constant $H_{F}$, the viscosity parameter ν, the geometrical parameter β, and the penalization parameter ε. Moreover, we give the evolution of the director field and velocity field of a rotating flow. Clearly, the annihilation time is much smaller than that obtained in reference [11]. Finally, the convergence of the numerical solution in time and space is analyzed.

This article is arranged as follows. Some necessary notations are given in Section 2. Some necessary hypotheses are given, and the fully discrete decoupling scheme is proposed in Section 3. In Section 4, we give a proof of a priori energy estimate for the algorithm, which provides an unconditional energy stability property. Finally, a number of numerical examples are provided to demonstrate the effects of the parameters and the performance of the scheme; the numerical accuracy of the proposed system in time and space also are given in Section 5. The conclusion is reported in Section 6.

## 2. Notations and Preliminaries

In this section, we present the essential notations and preliminaries which are necessary for further consideration.

### 2.1. Notations

As usual, $L^{p}\left(\Omega \right)\left(p\geq 1\right)$ denotes the space of *p*th-power integrable functions defined on Ω, and the norm is ${\left\|\cdot \right\|}_{L^{p}}={\left(\int_{\Omega }|\cdot {|}^{p}dx\right)}^{1/p}$ or ${\left\|\cdot \right\|}_{L^{\infty }}=$ess $\sup_{x\in \Omega }|\cdot |$. If p=2, the $L^{2}$ norm is ‖·‖, and we denote the inner product in $L^{2}$ by (·,·). For example, if $u\left(x\right),v\left(x\right)\in L^{2}$, $\left(u,v\right)=\int_{\Omega }u\left(x\right)v\left(x\right)dx$. For m as a non-negative integer, we denote the classical Sobolev spaces as

$$H^{m}\left(\Omega \right)=\left\{v\in L^{2};\partial^{k}v\in L^{2},\forall |k|\leq m\right\},$$

the corresponding norm

$${\left\|v\right\|}_{H^{m}\left(\Omega \right)}={\left(\sum_{0\leq |k|\leq m}{\left\|\partial^{k}v\right\|}^{2}\right)}^{\frac{1}{2}}.$$

Let $C_{0}^{\infty }$ be the space of infinitely times differentiable function with compact support on Ω. Then, $H_{0}^{m}\left(\Omega \right)$ is introduced as the closure of $C_{0}^{\infty }$ in $H^{m}\left(\Omega \right)$.

We now introduce the following function spaces in the context.

$$L_{0}^{2}=\left\{p:p\in L^{2}\left(\Omega \right),\int_{\Omega }p\left(x\right)dx=0\right\},$$

$$V=\{v\in C_{0}^{\infty }\left(\Omega \right),\nabla \cdot v=0\}.$$

Then, we define H and V as the closures of V in $L^{2}\left(\Omega \right)$ and $H^{1}\left(\Omega \right)$, respectively (see [17]).

$$H=\{u\in L^{2}\left(\Omega \right),\nabla \cdot u=0\;on\;\Omega ,u\cdot n=0\;on\;\partial \Omega \},$$

$$V=\{u\in H^{1}\left(\Omega \right),\nabla \cdot u=0\;on\;\Omega ,u=0\;on\;\partial \Omega \}.$$

### 2.2. A Penalized Ericksen–Leslie Model with Stretching Effect

A general penalty version of the Ericksen–Leslie model with stretching effect to enforce the sphere constraint reads as

(1) $$\begin{array}{ccc} \partial_{t}d+\left(u\cdot \nabla \right)d+\beta \left(\nabla u\right)d+\left(1+\beta \right){\left(\nabla u\right)}^{T}d+\gamma \left(f_{\varepsilon }\left(d\right)-\Delta d\right) & = & 0,in\Omega_{T},\\ \partial_{t}u+\left(u\cdot \nabla \right)u-\nu \Delta u+\nabla p+\lambda \nabla \cdot \left({\left(\nabla d\right)}^{T}\nabla d\right) \end{array}$$

(2) $$\begin{array}{ccc} +\lambda \nabla \cdot \left(\beta \left(f_{\varepsilon }\left(d\right)-\Delta d\right)d^{T}+\left(1+\beta \right)d{\left(f_{\varepsilon }\left(d\right)-\Delta d\right)}^{T}\right) & = & 0,in\Omega_{T}, \end{array}$$

(3) $$\begin{array}{ccc} \nabla \cdot u & = & 0,in\Omega_{T}, \end{array}$$

where $\Omega_{T}=\Omega \times \left(0,T\right]$, $\Omega \subset R^{M}\left(M=2,3\right)$ is a bounded open set with boundary ∂Ω, and T>0 is a fixed time, d (orientation of the molecules): ${\bar{\Omega }}_{T}\to R^{M}$, u (fluid velocity): ${\bar{\Omega }}_{T}\to R^{M}$, p (fluid pressure): ${\bar{\Omega }}_{T}\to R$, and $f_{\varepsilon }\left(d\right)$ is the penalty function related to the constraint ∣d∣=1; we define it by

(4) $$f_{\varepsilon }\left(d\right)=\left\{\begin{array}{c} \frac{1}{\varepsilon^{2}}\left(|d{|}^{2}-1\right)d\;if|d|\leq 1,\\ \frac{2}{\varepsilon^{2}}\left(|d|-1\right)\frac{d}{|d|}\;if|d|>1, \end{array}\right.$$

where ε>0 is the penalty parameter. The function $f_{\varepsilon }\left(d\right)$ is the gradient of the following scalar potential function $F_{\varepsilon }\left(d\right)$,

(5) $$F_{\varepsilon }\left(d\right)=\left\{\begin{array}{c} \frac{1}{4\varepsilon^{2}}{\left(|d{|}^{2}-1\right)}^{2}\;if|d|\leq 1,\\ \frac{1}{\varepsilon^{2}}{\left(|d|-1\right)}^{2}\;if|d|>1. \end{array}\right.$$

It is easy to verify that $\nabla_{d}F_{\varepsilon }\left(d\right)=f_{\varepsilon }\left(d\right)$. Furthermore, the parameters γ,ν, and λ>0 represent relaxation time, viscosity, and elasticity, respectively. Additionally, parameter β∈[−1,0] is a constant that determines the geometry of the molecule. For instance, when β=−1,−1/2,0, the molecules are rod-shaped, spherical, and disk-shaped [10,11,18], respectively.

To system (1)–(3), we add homogeneous Dirichlet conditions for the velocity field and homogeneous Neumann boundary conditions for the director field,

(6) $$u\left(x,t\right)=0,\partial_{n}d\left(x,t\right)=0\;\;for\;\left(x,t\right)\in \partial \Omega ,$$

and the initial conditions

(7) $$\;u\left(x,0\right)=u_{0}\left(x\right),d\left(x,0\right)=d_{0}\left(x\right)\;\;for\;x\in \Omega .$$

In order to better understand our proposed decoupling scheme, we hereby give an energy law for system (1)–(3), which is true under some regularity assumptions for d and u (see [10,13,19] for details). First, we give equations

$$\lambda \nabla \cdot \left({\left(\nabla d\right)}^{T}\nabla d\right)=\lambda \nabla \left(\frac{1}{2}|\nabla d{|}^{2}+F_{\varepsilon }\left(d\right)\right)-\lambda {\left(\nabla d\right)}^{T}\left(f_{\varepsilon }\left(d\right)-\Delta d\right),$$

and

$$\left[\left(u\cdot \nabla \right)d\right]\cdot \left(f_{\varepsilon }\left(d\right)-\Delta d\right)={\left(\nabla d\right)}^{T}\left(f_{\varepsilon }\left(d\right)-\Delta d\right)\cdot u.$$

Then, taking the inner product of (1) and (2) with $\lambda (f_{\varepsilon }\left(d\right)-\Delta d)$ and u, respectively, we have

$$\frac{d}{dt}E\left(u,d\right)+{\nu \|\nabla u\|}^{2}+\lambda \gamma {\left\|f_{\varepsilon }\left(d\right)-\Delta d\right\|}^{2}=0,$$

where

$$E\left(u,d\right)=\frac{1}{2}{\left\|u\right\|}^{2}+\frac{\lambda }{2}{\left\|\nabla d\right\|}^{2}+\lambda \int_{\Omega }f_{\varepsilon }\left(d\right),$$

here, $\frac{1}{2}{\left\|u\right\|}^{2}$ represents the kinetic energy, $\frac{\lambda }{2}{\left\|\nabla d\right\|}^{2}$ represents the elastic energy, $\lambda \int_{\Omega }F_{\varepsilon }\left(d\right)$ represents the penalty energy, and obviously, the total energy is E(u,d).

## 3. Hypotheses and the Fully Discrete Scheme

### 3.1. Hypotheses

We introduce the hypotheses that are required in the following.

**(H1)**  Let ∂Ω be a polygonal or polyhedral Lipschitz-continuous boundary.
**(H2)**  Let K represent any subregion after dividing $\bar{\Omega }$ into finite subregions, also called element domain. Additionally, its a bounded closed set with nonempty interior and piecewise smooth boundary. We use ${\left\{T_{h}\right\}}_{h>0}$ for all of K, so $\bar{\Omega }={⋃}_{K\in T_{h}}K$. In general, K is a triangle or quadrilateral in a two-dimensional space and a tetrahedron or hexahedron in a three-dimensional space.
**(H3)**  Assume that $\left(u_{0}\times d_{0}\right)\in H\times H^{1}$ with $|d_{0}|=1$ a.e. in Ω.
**(H4)**  Suppose that $D_{h}\subset H^{1}\left(\Omega \right),V_{h}\subset H_{0}^{1}\left(\Omega \right)$ and $P_{h}\subset H^{1}\left(\Omega \right)⋂L_{0}^{2}\left(\Omega \right)$ are a conformed finite element space associated with $T_{h}$.
**(H5)**  Let $P_{1}\left(K\right)$ denote the set of linear polynomials on K. Under Hypotheses 4, the space of continuous, piecewise polynomial functions associated to $T_{h}$ are denoted as follows:
  $$X_{h}=\left\{x_{h}\in C^{0}\left(\bar{\Omega }\right),x_{h}{|}_{K}\in P_{1}\left(K\right),\forall K\in T_{h}\right\},$$
  and denote the space of piecewise constant function as
  $$Y_{h}=\left\{y_{h}\in L^{\infty }\left(\Omega \right),y_{h}{|}_{K}\in R,\forall K\in T_{h}\right\},$$
  where $Y_{h}$ is the classical $R_{0}\left(orP_{0}\right)$ space.
**(H6)**  The triangulation of Ω and the discrete spaces satisfy ([5,6,11]):
  (a.) The approximation properties:
  (8) $$\|d-I_{h}d\|\leq C_{1}h{\left\|d\right\|}_{H^{1}}\;\forall d\in H^{1}\left(\Omega \right).$$
  (b.) The stability properties:
  (9) $$\|I_{h}{d\|}_{H^{1}\left(\Omega \right)}\leq C_{2}{\left\|d\right\|}_{H^{1}\left(\Omega \right)}\;\forall d\in H^{1}\left(\Omega \right),$$
  (10) $$\|I_{h}{d\|}_{L^{\infty }\left(\Omega \right)}\leq C_{3}{\left\|d\right\|}_{L^{\infty }\left(\Omega \right)}\;\forall d\in L^{\infty }\left(\Omega \right),$$
  where $I_{h}$ is an interpolation operator into $D_{h}$, and $C_{1},C_{2},$ and $C_{3}$ are constant independents of *h*.

In this paper, we, respectively, choose $D_{h}=X_{h},V_{h}=X_{h}⋂H_{0}^{1}\left(\Omega \right)$, and $P_{h}=X_{h}⋂L_{0}^{2}\left(\Omega \right)$ as the approximate spaces of direction, velocity, and pressure. We choose discontinuous finite element space $W_{h}=Y_{h}$ as the approximate space of an auxiliary variable. The finite element spaces that we choose for velocity and pressure do not satisfy the discrete inf–sup condition

(11) $$\begin{array}{c} \|p_{h}{\|}_{0}\leq \alpha \underset{v_{h}\in V_{h}\setminus \left\{0\right\}}{\sup}\frac{\left(p_{h},\nabla \cdot v_{h}\right)}{\|v_{h}{\|}_{H^{1}\left(\Omega \right)}}\;\forall p_{h}\in P_{h}, \end{array}$$

for α>0 independent of h.

### 3.2. The Fully Discrete Scheme

Here, we use the nonincremental pressure-correction method to obtain the fully discrete scheme of system (1)–(3).

Initialization Let $\left(d_{h}^{0},u_{h}^{0},p_{h}^{0}\right)\in D_{h}\times V_{h}\times P_{h}$ be a suitable approximation of $(d_{0},u_{0},$$p_{0})$

(12) $$\left\{\begin{array}{c} d_{h}^{0}=I_{h}d_{0},\\ \left(u_{h}^{0},{\tilde{u}}_{h}\right)+\left(\nabla p_{h}^{0},{\tilde{u}}_{h}\right)=\left(u_{0},{\tilde{u}}_{h}\right),\\ \left(\nabla \cdot u_{h}^{0},q_{h}\right)+j\left(p_{h}^{0},q_{h}\right)=0. \end{array}\right.$$

for all ${\tilde{u}}_{h}\in V_{h},q_{h}\in P_{h}$.

The stabilization term j(p,q) can be defined as

(13) $$j\left(p,q\right)=\frac{S}{\nu }\left(p-\Pi_{h}p,q-\Pi_{h}q\right),$$

where S is an algorithmic constant and $\Pi_{h}$ is a standard $L^{2}$-projection operator onto $Y_{h}$. This stabilization term is also used in [20].

Step(n+1) We discretize system (1)–(3) with uniform time steps, $0=t_{0}<t_{1}<\cdots <t_{N}=T$ with Δt=T/N. Let $\left(d_{h}^{n},u_{h}^{n},p_{h}^{n}\right)\in D_{h}\times V_{h}\times P_{h}$ be given. For n+1, n≥0, perform the following steps:

**(1)**  For all $\left({\tilde{d}}_{h},{\tilde{\omega }}_{h}\right)\in D_{h}\times W_{h}$, find the numerical approximation $\left(d_{h}^{n+1},\omega_{h}^{n+1}\right)\in D_{h}\times W_{h}$ satisfying
  (14) $$\left\{\begin{array}{c} \left(\frac{d_{h}^{n+1}-d_{h}^{n}}{\Delta t},{\tilde{\omega }}_{h}\right)+\left(\left(u_{h1}\cdot \nabla \right)d_{h}^{n},{\tilde{\omega }}_{h}\right)-\beta \left(\nabla \cdot \left({\tilde{\omega }}_{h}{\left(d_{h}^{n}\right)}^{T}\right),u_{h2}\right)\\ -\left(1+\beta \right)\left(\nabla \cdot \left(d_{h}^{n}{\left({\tilde{\omega }}_{h}\right)}^{T}\right),u_{h3}\right)+\gamma \left(\omega_{h}^{n+1},{\tilde{\omega }}_{h}\right)=0,\\ \left(\nabla d_{h}^{n+1},\nabla {\tilde{d}}_{h}\right)+\left(f_{\varepsilon }\left(d_{h}^{n}\right),{\tilde{d}}_{h}\right)+\frac{H_{F}}{2\varepsilon^{2}}\left(d_{h}^{n+1}-d_{h}^{n},{\tilde{d}}_{h}\right)-\left(\omega_{h}^{n+1},{\tilde{d}}_{h}\right)=0, \end{array}\right.$$
  herein,
  (15) $$\begin{array}{ccc} & & u_{h1}=u_{h}^{n}-\Delta t\nabla p_{h}^{n}+3\lambda \Delta t{\left(\nabla d_{h}^{n}\right)}^{T}\omega_{h}^{n+1},\\ & & u_{h2}=u_{h}^{n}-\Delta t\nabla p_{h}^{n}-3\lambda \beta \Delta t\nabla \cdot \left(\omega_{h}^{n+1}{\left(d_{h}^{n}\right)}^{T}\right),\\ & & u_{h3}=u_{h}^{n}-\Delta t\nabla p_{h}^{n}-3\lambda \left(1+\beta \right)\Delta t\nabla \cdot \left(d_{h}^{n}{\left(\omega_{h}^{n+1}\right)}^{T}\right), \end{array}$$
  and $H_{F}>0$ is a bound of the $L^{\infty }$-norm of the Hessian matrix associated to $F_{\varepsilon }\left(d\right)$. For instance,
  (16) $$H_{F}:={\left(M3^{2}+\left(M^{2}-M\right)2^{2}\right)}^{\frac{1}{2}}$$
  with M being the space dimension ([6]).
**(2)**  By using the nonincremental pressure-correction method for all ${\tilde{u}}_{h}\in V_{h}$, find $u_{h}^{n+1}\in V_{h}$ satisfying
  (17) $$\begin{array}{ccc} & & \left(\frac{u_{h}^{n+1}-u_{h}^{n}}{\Delta t},{\tilde{u}}_{h}\right)+\nu \left(\nabla u_{h}^{n+1},\nabla {\tilde{u}}_{h}\right)+c\left(u_{h}^{n},u_{h}^{n+1},{\tilde{u}}_{h}\right)\\ & & -\lambda \left({\left(\nabla d_{h}^{n}\right)}^{T}\omega_{h}^{n+1},{\tilde{u}}_{h}\right)+\lambda \beta \left(\nabla \cdot \left(\omega_{h}^{n+1}{\left(d_{h}^{n}\right)}^{T}\right),{\tilde{u}}_{h}\right)\\ & & +\lambda \left(1+\beta \right)\left(\nabla \cdot \left(d_{h}^{n}{\left(\omega_{h}^{n+1}\right)}^{T}\right),{\tilde{u}}_{h}\right)+\left(\nabla p_{h}^{n},{\tilde{u}}_{h}\right)=0. \end{array}$$
  We set the trilinear convective term
  (18) $$c\left(u_{h}^{n},u_{h}^{n+1},{\tilde{u}}_{h}\right)=\left(\left(u_{h}^{n}\cdot \nabla \right)u_{h}^{n+1},{\tilde{u}}_{h}\right)+\frac{1}{2}\left(\nabla \cdot u_{h}^{n},u_{h}^{n+1}\cdot {\tilde{u}}_{h}\right)$$
  has skew-symmetry, so $c(u_{h}^{n},u_{h}^{n+1},u_{h}^{n+1})=0$.
**(3)**  For all $q_{h}\in P_{h}$, find $p_{h}^{n+1}\in P_{h}$ satisfying
  (19) $$\Delta t\left(\nabla p_{h}^{n+1},\nabla q_{h}\right)+j\left(p_{h}^{n+1},q_{h}\right)=-\left(\nabla \cdot u_{h}^{n+1},q_{h}\right).$$

Since scheme (14)–(19) is linear, we can easily prove its existence and the uniqueness of the solution by using the same techniques as in [6].

## 4. Energy Estimate

In this section, we give the proof about energy estimates for schemes (14)–(19). First of all, we give an inequality in the following, the proof of which already exists in [6].

(20) $$\lambda \left((f_{\varepsilon }\left(d_{h}^{n}\right)+\frac{H_{F}}{2\varepsilon^{2}}\left(d_{h}^{n+1}-d_{h}^{n}\right),\left(d_{h}^{n+1}-d_{h}^{n}\right)\right)\geq \lambda \int_{\Omega }\left(F_{\varepsilon }\left(d_{h}^{n+1}\right)-F_{\varepsilon }\left(d_{h}^{n}\right)\right).$$

**Theorem** **1.** *Assume that the hypotheses in Section 3.1 are satisfied. In addition, let*

(21) $${\hat{u}}_{h}^{n+1}=u_{h}^{n+1}-\Delta t\nabla p_{h}^{n+1}.$$

*Then, the numerical solutions $(d_{h}^{n+1},\omega_{h}^{n+1},u_{h}^{n+1},$ and $p_{h}^{n+1})$ of the schemes (14)–(19) have the property*

(22) $$E\left({\hat{u}}_{h}^{n+1},d_{h}^{n+1}\right)-E\left({\hat{u}}_{h}^{n},d_{h}^{n}\right)\leq 0.$$

**Proof.**  We choose ${\tilde{\omega }}_{h}=\lambda \Delta t\omega_{h}^{n+1}$ and ${\tilde{d}}_{h}=\lambda \left(d_{h}^{n+1}-d_{h}^{n}\right)$ in (14), and take into account (20), obtaining

(23) $$\begin{array}{ccc} & & \frac{\lambda }{2}\|\nabla d_{h}^{n+1}{\|}^{2}-\frac{\lambda }{2}\|\nabla d_{h}^{n}{\|}^{2}+\frac{\lambda }{2}\|\nabla d_{h}^{n+1}-\nabla d_{h}^{n}{\|}^{2}+\gamma \lambda \Delta t{\left\|\omega_{h}^{n+1}\right\|}^{2}\\ & & +\lambda \int_{\Omega }\left(F_{\varepsilon }\left(d_{h}^{n+1}\right)-F_{\varepsilon }\left(d_{h}^{n}\right)\right)+\lambda \Delta t\left(\left(u_{h1}\cdot \nabla \right)d_{h}^{n},\omega_{h}^{n+1}\right)\\ & & +\beta \lambda \Delta t\left(\left(\nabla u_{h2}\right)d_{h}^{n},\omega_{h}^{n+1}\right)+\left(1+\beta \right)\lambda \Delta t\left({\left(\nabla u_{h3}\right)}^{T}d_{h}^{n},\omega_{h}^{n+1}\right)\leq 0. \end{array}$$

Moreover, selecting ${\tilde{u}}_{h}=\Delta tu_{h}^{n+1}$ in (17) and defining ${\bar{u}}_{h}=\frac{u_{h1}+u_{h2}+u_{h3}}{3}$, it follows that

(24) $$\frac{1}{2}\|u_{h}^{n+1}{\|}^{2}-\frac{1}{2}\|{\bar{u}}_{h}{\|}^{2}+\frac{1}{2}\|{\bar{u}}_{h}-u_{h}^{n+1}{\|}^{2}+\nu \Delta t{\left\|\nabla u_{h}^{n+1}\right\|}^{2}=0.$$

Next, choosing $q_{h}=p_{h}^{n+1}$ in (19) and by using the equality (21), we have

(25) $$j\left(p_{h}^{n+1},p_{h}^{n+1}\right)=\left(u_{h}^{n+1}-\Delta t\nabla p_{h}^{n+1},\nabla p_{h}^{n+1}\right)=\left({\hat{u}}_{h}^{n+1},\nabla p_{h}^{n+1}\right),$$

therefore, taking the inner product of both sides of (21) with itself, we obtain

(26) $$\frac{1}{2}\|{\hat{u}}_{h}^{n+1}{\|}^{2}-\frac{1}{2}\|u_{h}^{n+1}{\|}^{2}+\frac{1}{2}{\left\|u_{h}^{n+1}-{\hat{u}}_{h}^{n+1}\right\|}^{2}+\Delta tj\left(p_{h}^{n+1},p_{h}^{n+1}\right)=0.$$

Hence, we obtain, by adding Equations (24) and (26),

(27) $$\begin{array}{ccc} & & \frac{1}{2}\|{\hat{u}}_{h}^{n+1}{\|}^{2}-\frac{1}{2}\|{\bar{u}}_{h}{\|}^{2}+\frac{1}{2}\|{\bar{u}}_{h}-u_{h}^{n+1}{\|}^{2}+\nu \Delta t{\left\|\nabla u_{h}^{n+1}\right\|}^{2}\\ & & +\frac{\Delta t^{2}}{2}{\left\|\nabla p_{h}^{n+1}\right\|}^{2}+\Delta tj\left(p_{h}^{n+1},p_{h}^{n+1}\right)=0. \end{array}$$

In addition, taking the inner product of both sides of $u_{h1},u_{h2},$ and $u_{h3}$ in (15) with $u_{h1},u_{h2},$ and $u_{h3}$, respectively, we have

(28) $$\frac{1}{6}\|u_{h1}{\|}^{2}-\frac{1}{6}\|{\hat{u}}_{h}^{n}{\|}^{2}+\frac{1}{6}{\left\|u_{h1}-{\hat{u}}_{h}^{n}\right\|}^{2}-\lambda \Delta t\left({\left(\nabla d_{h}^{n}\right)}^{T}\omega_{h}^{n+1},u_{h1}\right)=0,$$

(29) $$\frac{1}{6}\|u_{h2}{\|}^{2}-\frac{1}{6}\|{\hat{u}}_{h}^{n}{\|}^{2}+\frac{1}{6}{\left\|u_{h2}-{\hat{u}}_{h}^{n}\right\|}^{2}+\beta \lambda \Delta t\left(\nabla \cdot \left(\omega_{h}^{n+1}{\left(d_{h}^{n}\right)}^{T}\right),u_{h2}\right)=0,$$

(30) $$\frac{1}{6}\|u_{h3}{\|}^{2}-\frac{1}{6}\|{\hat{u}}_{h}^{n}{\|}^{2}+\frac{1}{6}{\left\|u_{h3}-{\hat{u}}_{h}^{n}\right\|}^{2}+\left(1+\beta \right)\lambda \Delta t\left(\nabla \cdot \left(d_{h}^{n}{\left(\omega_{h}^{n+1}\right)}^{T}\right),u_{h3}\right)=0.$$

From the definition of ${\bar{u}}_{h}$, we have

(31) $$\frac{{\bar{u}}_{h}-u_{h1}}{3}+\frac{{\bar{u}}_{h}-u_{h2}}{3}+\frac{{\bar{u}}_{h}-u_{h3}}{3}=0,$$

therefore,

(32) $$\begin{array}{ccc} & & \frac{1}{2}{\left\|{\bar{u}}_{h}\right\|}^{2}-\frac{1}{6}\left(\|u_{h1}{\|}^{2}+\|u_{h2}{\|}^{2}+{\left\|u_{h3}\right\|}^{2}\right)\\ & & +\frac{1}{6}\left(\|u_{h1}-{\bar{u}}_{h}{\|}^{2}+\|u_{h2}-{\bar{u}}_{h}{\|}^{2}+{\left\|u_{h3}-{\bar{u}}_{h}\right\|}^{2}\right)=0. \end{array}$$

Adding (23), (27)–(30), and (32) implies that

(33) $$\begin{array}{ccc} & & E\left({\hat{u}}_{h}^{n+1},d_{h}^{n+1}\right)-E\left({\hat{u}}_{h}^{n},d_{h}^{n}\right)+\frac{\lambda }{2}\|\nabla d_{h}^{n+1}-\nabla d_{h}^{n}{\|}^{2}+\gamma \lambda \Delta t{\left\|\omega_{h}^{n+1}\right\|}^{2}\\ & & +\frac{1}{2}\|{\bar{u}}_{h}-u_{h}^{n+1}{\|}^{2}+\nu \Delta t\|\nabla u_{h}^{n+1}{\|}^{2}+\frac{\Delta t^{2}}{2}{\left\|\nabla p_{h}^{n+1}\right\|}^{2}+\Delta tj\left(p_{h}^{n+1},p_{h}^{n+1}\right)\\ & & +\frac{1}{6}\left(\right\|u_{h1}-{\hat{u}}_{h}^{n}{\|}^{2}+\|u_{h2}-{\hat{u}}_{h}^{n}{\|}^{2}+\|u_{h3}-{\hat{u}}_{h}^{n}{\|}^{2})\\ & & +\frac{1}{6}\left(\|u_{h1}-{\bar{u}}_{h}{\|}^{2}+\|u_{h2}-{\bar{u}}_{h}{\|}^{2}+{\left\|u_{h3}-{\bar{u}}_{h}\right\|}^{2}\right)\leq 0, \end{array}$$

which implies the assertion (22). □

Theorem 1 is a result concerning a local discrete energy estimate. Then, we give a global energy estimate about time for schemes (12)–(19) in the following theorem.

**Theorem** **2.** *Assume that the hypotheses in Section 3.1 are satisfied. For any n∈[0,N], the numerical solutions $(d_{h}^{n},\omega_{h}^{n},$ and $u_{h}^{n})$ of the schemes (14)–(19) have the property*

(34) $$\max_{m\in [0,N-1]}\{E\left(u_{h}^{m+1},d_{h}^{m+1}\right)+\Delta t\sum_{n=0}^{m}(\nu \|\nabla u_{h}^{n+1}{\|}^{2}+\lambda \gamma \|\omega_{h}^{n+1}{\|}^{2})\}\leq E\left(u_{h}^{0},d_{h}^{0}\right),$$

*where $\left(u_{h}^{0}\;and\;d_{h}^{0}\right)$ are defined in (12). Additionally, if for some constant K>0, (h,ε) satisfy h/ε≤K, then there exists a constant $C_{0}>0$ such that*

(35) $$\max_{m\in [0,N-1]}\{E\left(u_{h}^{m+1},d_{h}^{m+1}\right)+\Delta t\sum_{n=0}^{m}(\nu \|\nabla u_{h}^{n+1}{\|}^{2}+\lambda \gamma \|\omega_{h}^{n+1}{\|}^{2})\}\leq C_{0}.$$

**Proof.**  The proof of (34) follows easily from Theorem 1 and by summing over n. Using the same method as Lemma 4.4 in [6], we can prove that $E\left(u_{h}^{0},d_{h}^{0}\right)\leq C_{0}$. Then, we can easily obtain (35). □

## 5. Numerical Experiments

In this section, we present a numerical example to carry out a sensitivity study of schemes (12)–(19). More precisely, we give details to take care of the relation among the stabilization constant $H_{F}$ defined in (16), the viscosity parameter ν, the geometrical parameter β, and the penalization parameter ε. Then, we consider a rotating flow and give the evolution of the director field and velocity field for the annihilation of two and four singularities. At the end of this paper, we investigate the numerical accuracy of the proposed system in space and time.

The numerical solutions are implemented by FreeFem++ [21] and Matlab.

### 5.1. Annihilation of Singularities

We consider the initial conditions of the nematic liquid crystal with stretching effect (1)–(3) are

$$\begin{array}{cc} & u_{0}=0,\\ & d_{0}=\frac{\tilde{d}}{\sqrt{|\tilde{d}{|}^{2}+\varepsilon^{2}}}, \end{array}$$

where $\tilde{d}=\left(x^{2}+y^{2}-0.25,y\right)$. This example was also used to research nematic liquid crystal in [5,6,10,11,13,22]. In this experiment, we choose computational domain Ω=(−1,1)×(−1,1), time step size Δt=0.001, and use a 32×32 grid in the computation.

**Figure 1:**
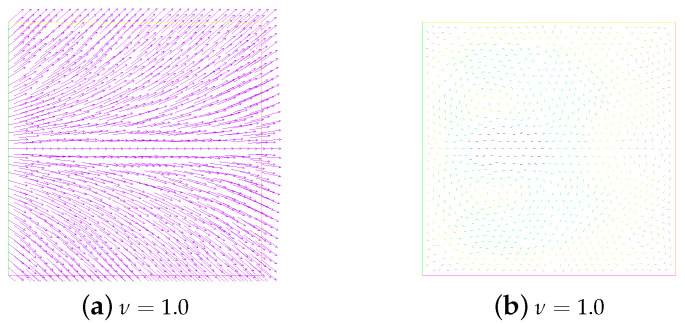

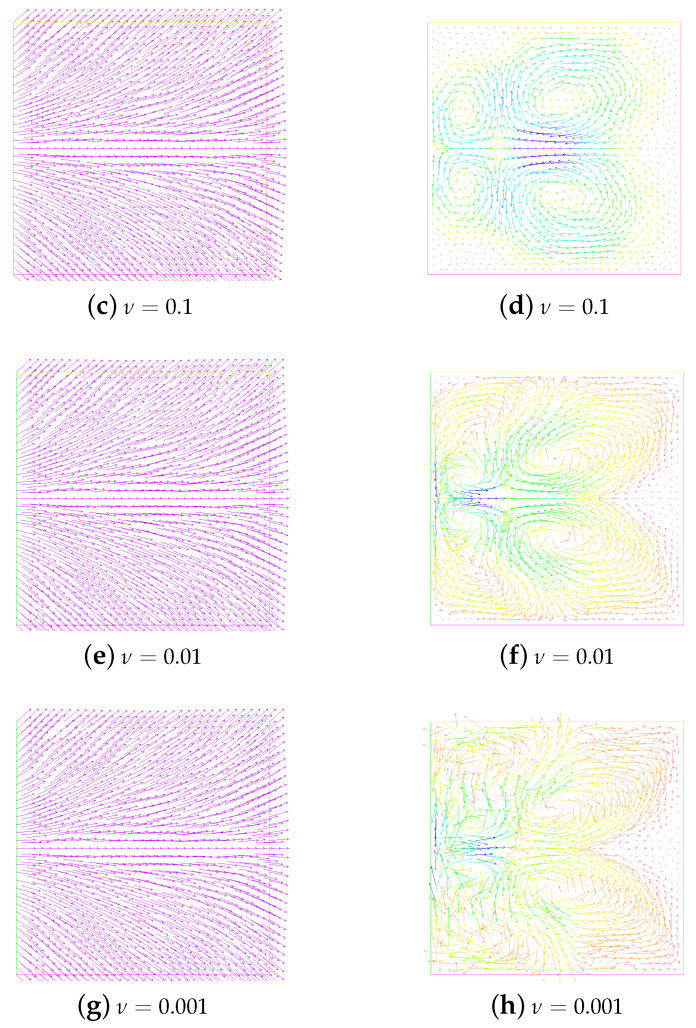
Evolution of the director field (the first column) and the velocity field (the second column) for the annihilation of two singularities at ν=1.0,0.1,0.01, and 0.001. Here, β=−1, ϵ=0.05, T = 1, and M=2.

**Figure 2:**
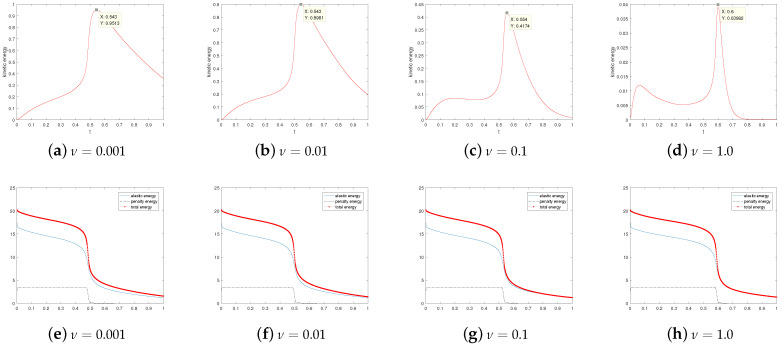
Evolution of the energies for the annihilation of two singularities at ν=0.001,0.01,0.1, and 1.0. Here, β=−1, ϵ=0.05, T = 1, and M=2.

First of all, let β=−1 and ε=0.05. We research the stability, annihilation time, and energies of two singularities at M=0,1,2,3 under different viscosity coefficient ν; see Table 1. We are concerned with the dependence of stability on the parameters *M* and ν. In particular, we present snapshots of the director and velocity fields at M=2 and the time T=1.0 in Figure 1. As can be seen from Figure 1, when the numerical solution is in a stable state, different ν values have no great influence on the director field, where the stability refers to the ability of fluid motion of a certain form to recover its original form after initial disturbance. However, it is obvious that the velocity field is somewhat chaotic when ν=0.001, while the trend of the velocity field becomes regular when ν gradually increases. Especially at ν=1.0, the velocity field has largely quieted down. A, we also show the behavior of the energies in Figure 2. We can find that with the annihilation of the singularity, the energy decreases rapidly, which is consistent with the results obtained in [10].

Secondly, we investigate the dependence of stability on parameters $H_{F}$ and β. We take ν=1.0, and ε=0.05 and vary β=0,−0.5,−1, and M=0,1,2,3. The results are shown in Table 2.

**Figure 3:**
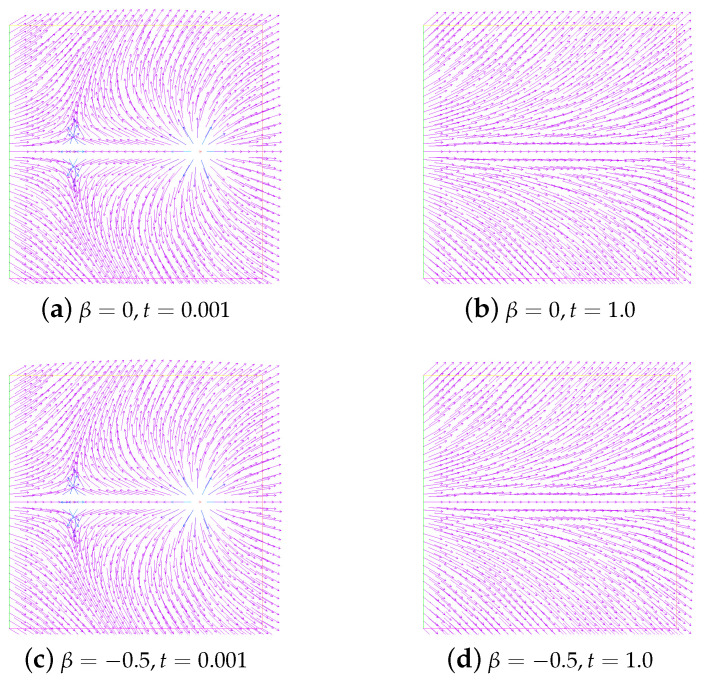

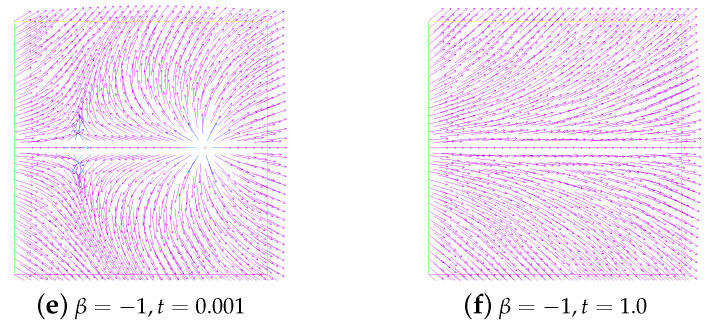
Snapshots of the director field in the initial state and the stable state with different β. Here, ν=1.0,ε=0.05, and M=2.

In order to more intuitively observe the influence of different β values on the annihilation, we specially give snapshots of the director field in the initial state and the stable state in Figure 3. We do not find any significant difference between these figures with these different β values.

Then, we investigate the dependence of stability on parameters $H_{F}$ and ε. We take ν=1.0 and β=−1 and vary ε=0.1,0.05,0.01, and 0.001 and M=0,1,2,3. The results are shown in Table 3. The results are similar to those presented in Table 4 of [11]. It is found that when ε=0.1, and 0.05, the annihilation time becomes longer and longer with the increase in M, and the maximum value of the corresponding kinetic energy becomes smaller and smaller. Even when ε=0.01 and 0.001, where there is no longer annihilation, this rule can still be followed.

**Figure 4:**
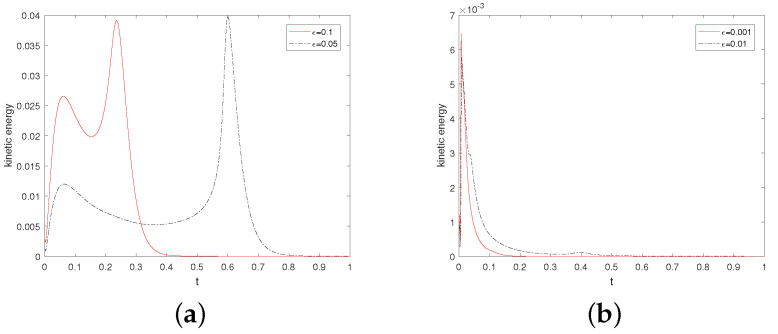
Evolution in time of the kinetic energy for ε=0.1,0.05,0.01, and 0.001. Here, ν=1.0,β=−1, and M=2.

Figure 4 shows that evolution in time of the kinetic energy for different ε values when M=2. By observation, we find that the kinetic energy behavior is obviously different under the conditions that singularities can annihilate (a) or not annihilate (b). As noted in references [6,11], a possible explanation for this behavior is that the velocity field generated by the elastic tensor is insufficient to move the singularity though the convective term in the director equation. Additionally, especially at ε=0.01, and 0.001, the kinetic energy goes down to zero at the beginning. One might think that if the kinetic energy associated with the velocity field is large enough to move the singularities, then they will move towards each other until annihilation.

**Figure 5:**
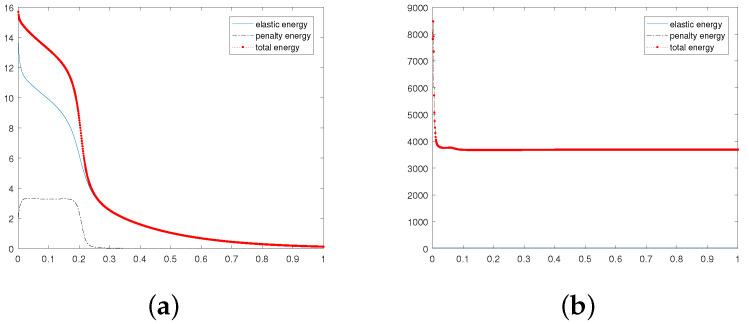
Evolution in time of energies for ε=0.1,0.001,ν=1.0,β=−1, and M=1.

Furthermore, to visualize the difference in energy in the stable state and unstable state, we present Figure 5.

**Figure 6:**
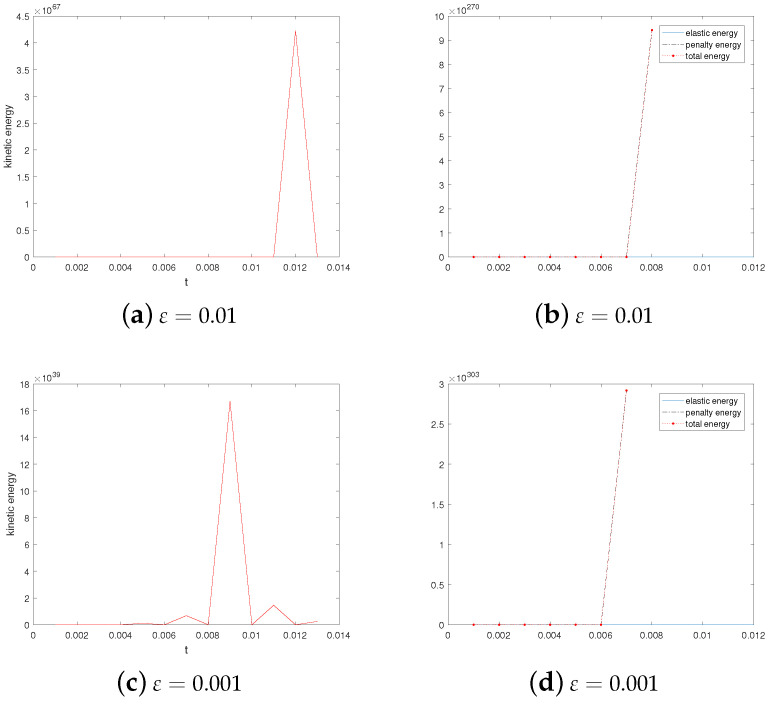
Evolution in time of energies for ε=0.01, and 0.001. Here, ν=1.0,β=−1, and M=0.

**Figure 7:**
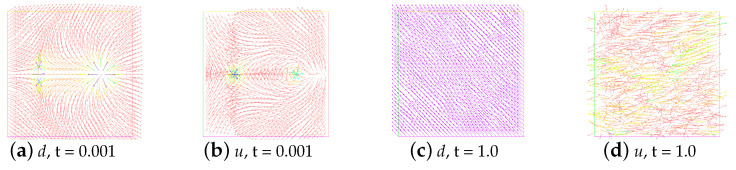
Snapshots of the unstable state at time t=0.001 and t=1.0. Here, ε=0.001,ν=1,β=−1, and M=0.

**Figure 8:**
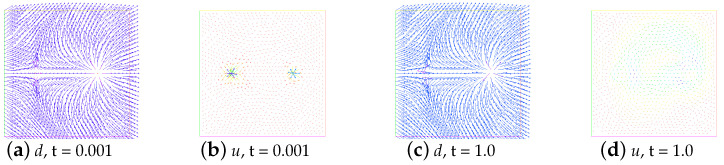
Snapshots of no annihilation of director field (**a**,**c**) and velocity field (**b**,**d**) at times t=0.001 and t=1.0. Here, ε=0.001,ν=1,β=−1, and M=1.

In addition, in Figure 6, we show the evolution in time of energy for the unstable state. Obviously, they are completely different from the evolution of the energy for the stable state. The snapshots of the unstable state (ε=0.001, and M=0) and the no annihilation (ε=0.001, and M=1) at t=0.001 and t=1.0 are given in Figure 7 and Figure 8, respectively.

**Figure 9:**
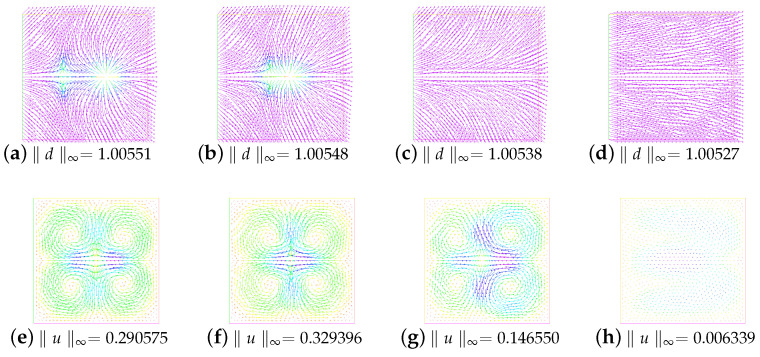
The snapshots of the director and the velocity field at times t=0.1,0.2,0.3, and 1.0. Here, we choose the parameters ε=0.1,β=−1,ν=1.0,M=2,λ=1.0,γ=1.0, and Δt=0.001.

Lastly, we give the snapshots of the director and the velocity field displayed at times t=0.1,0.2,0.3, and 1.0 in Figure 9. Here, we choose the parameters ε=0.1,β=−1,ν=1.0,M=2,λ=1.0,γ=1.0, and Δt=0.001.

### 5.2. The Behavior under Rotating Flow

**Figure 10:**
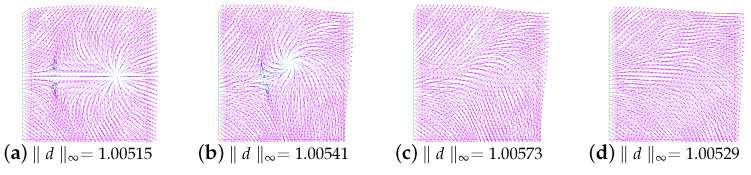
The snapshots of the director field of two singularities for the rotating flow at times t=0.001,0.4,0.6, and 1.0.

**Figure 11:**
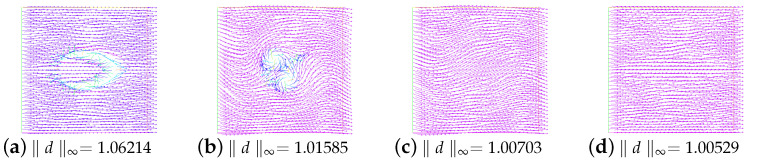
The snapshots of the director field of four singularities for the rotating flow at times t=0.001,0.1,0.2, and 1.0.

In this example, we consider a rotating flow in a square domain Ω=(−1,1)×(−1,1). The initial director field of two singularities is the same as Example 5.1. The initial director field of four singularities is the same as Section 4.2.2 in [11]. The initial velocity field is $u_{0}=\left(-20y,20x\right)$. We present the snapshots of the director field for the annihilation of two singularities at times t=0.001,0.4,0.6, and 1.0 and four singularities at times t=0.001,0.1,0.2, and 1.0 (see Figure 10 and Figure 11). Here, we choose the parameters ε=0.05,β=−1,ν=1.0,M=2,λ=1.0, and γ=1.0. Additionally, Δt=0.001. Clearly, the annihilation times are around t=0.555 and t=0.160, respectively. They are all smaller than those obtained in [11].

### 5.3. Convergence Rate

In this subsection, we consider that the initial conditions of the nematic liquid crystal with stretching effect (1)–(3) are

$$\begin{array}{cc} & u_{0}=0,\\ & d_{0}=\left(\sin\left(a\right),\cos\left(a\right)\right), \end{array}$$

where $a=\pi {\left(x^{2}+y^{2}\right)}^{2}$. Here, we choose computational domain $\Omega =\left(0,1\right)\times \left(-\frac{1}{2},\frac{1}{2}\right)$ and parameters ε=0.05,β=−1,ν=1.0,λ=1.0, and γ=1.0,M=2. To measure the convergence rate, we use the following equations:

$$\begin{array}{cc} & r_{L^{2},\Delta t}=\log_{2}\left(\frac{\|v_{h}^{\Delta t}-v_{h}^{2\Delta t}\|}{\|v_{h}^{\Delta t/2}-v_{h}^{\Delta t}\|}\right),r_{H^{1},\Delta t_{i}}=\log_{2}\left(\frac{\|v_{h}^{\Delta t}-v_{h}^{2\Delta t}{\|}_{H^{1}\left(\Omega \right)}}{\|v_{h}^{\Delta t/2}-v_{h}^{\Delta t}{\|}_{H^{1}\left(\Omega \right)}}\right),\\ & r_{L^{2},h}=\log_{2}\left(\frac{\|v_{h}^{\Delta t}-v_{2h}^{\Delta t}\|}{\|v_{h/2}^{\Delta t}-v_{h}^{\Delta t}\|}\right),r_{H^{1},h}=\log_{2}\left(\frac{\|v_{h}^{\Delta t}-v_{2h}^{\Delta t}{\|}_{H^{1}\left(\Omega \right)}}{\|v_{h/2}^{\Delta t}-v_{h}^{\Delta t}{\|}_{H^{1}\left(\Omega \right)}}\right). \end{array}$$

Here, v can be u,d, and *p*.

**Figure 12:**
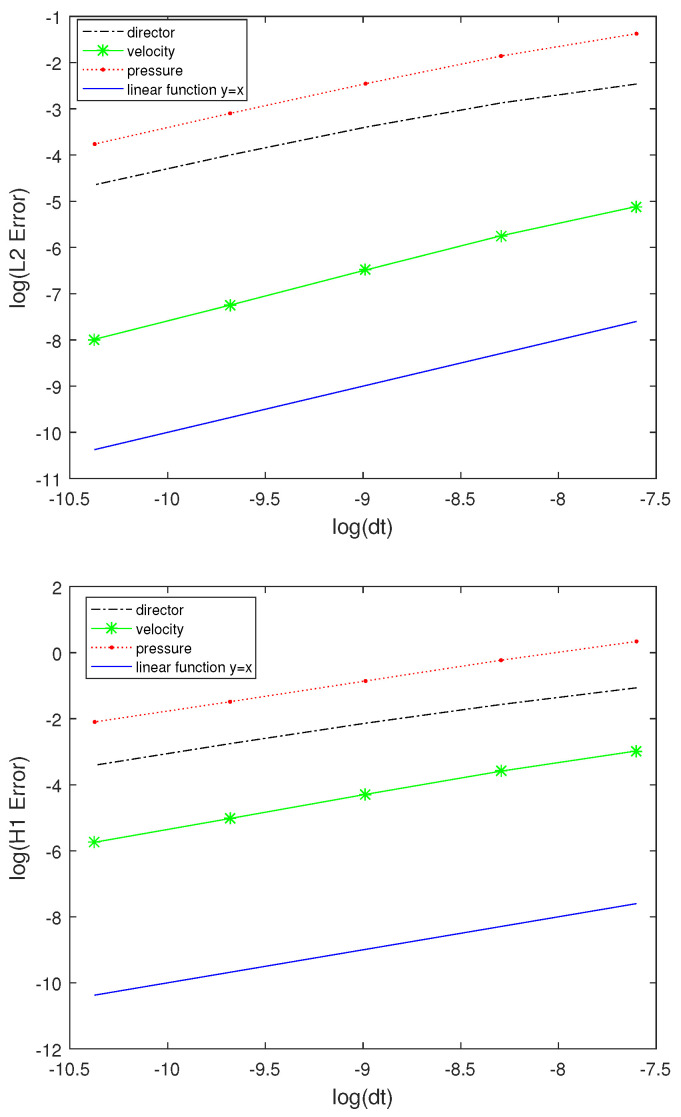
The error in time in $L^{2}-norm$ and $H^{1}-norm$.

In Figure 12 and Table 4, we present the time errors and convergence rates for the director, velocity, and pressure measured in the $L^{2}-norm$ and $H^{1}-norm$ at the final time T=0.1, respectively. The time error for the director, velocity, and pressure in the $L^{2}-norm$ and $H^{1}-norm$ are of O(Δt), respectively. Here, we run the code with the spatial mesh size: 64×64 and time steps $\Delta t=10^{-3},5\times 10^{-4},2.5\times 10^{-4},1.25\times 10^{-4},$ and $6.25\times 10^{-5}$, respectively.

**Figure 13:**
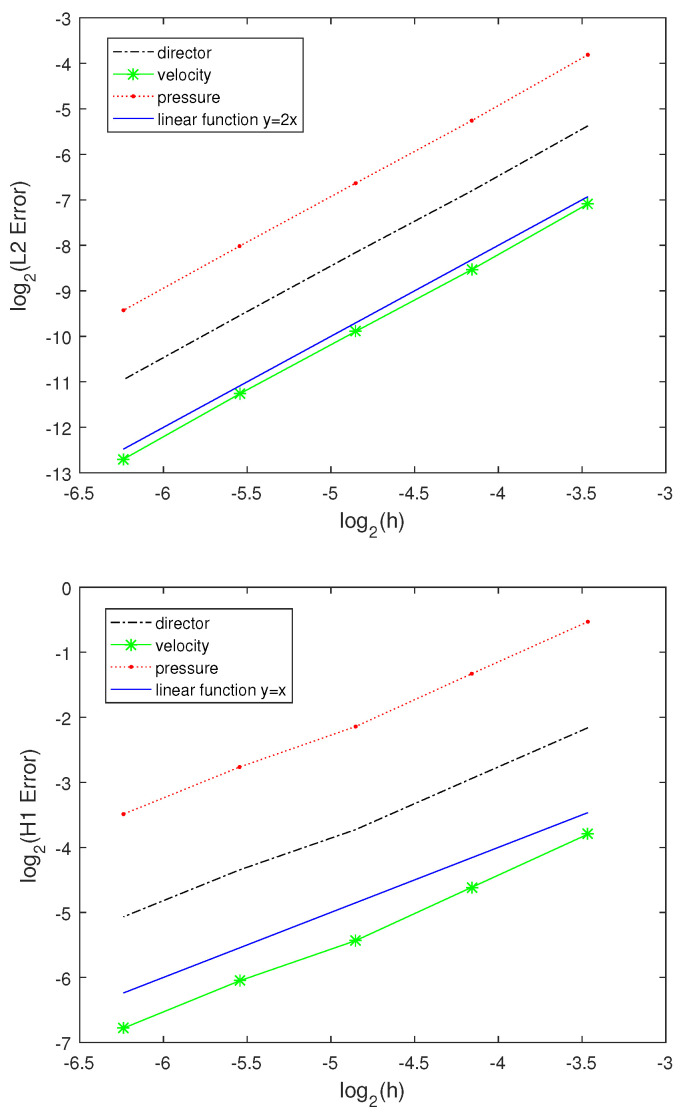
Behavior of the space error in the $L^{2}-norm$ and $H^{1}-norm$.

In Figure 13 and Table 5, we present the space errors and space convergence rates for the director, velocity, and pressure measured in the $L^{2}-norm$ and $H^{1}-norm$ at the final time T=0.1. The error for the director, velocity, and pressure in the $L^{2}-norm$ and $H^{1}-norm$ are of $O(h^{2})$ and O(h), respectively. Compared with the result obtained in [6], ours is obviously better. Here, we run the code with the spatial mesh sizes: 16×16,32×32,64×64,128×128, and 256×256 and time step Δt=0.001, respectively.

**Remark** **1.** *Compared with [6], which used the same method as this paper to research the Ericksen–Leslie model without stretching effect, the time and space convergence rates obtained in this paper are better.*

## 6. Conclusions

In this paper, a new decoupling scheme based on the nonincremental pressure-correction projection method was proposed to approximate the penalized Ericksen–Leslie model with stretching effect. The scheme is a linear and unconditionally stable system. One bright spot is that equal low-order finite element spaces were used for our scheme. That is, P1−P1−P1 finite element spaces were used for the director, velocity, and pressure.

In our numerical experiment, the sensitivity of the viscosity parameter ν, the geometrical parameter β, the penalization parameter ε, and the stabilization constant $H_{F}$ in the proposed scheme were studied. It was found that the sensitivity of singularity annihilation to parameters β,ε, and $H_{F}$ is very consistent with the result in [11]. Notably, we studied the effect of different viscosity coefficients on the annihilation and found that our method works for different ν (related to the Reynolds number). However, when ν=0.001, the velocity field looks messy near the singularity region. We will consider how to overcome this problem in future studies. In addition, experiments on the annihilation of two singularities and four singularities in a rotating flow were performed. What is gratifying is that by comparing with the existing methods, the results we obtain are better than those in [11]. Furthermore, we verified the numerical accuracy in time and space.

## Figures and Tables

**Table 1:** The stability, annihilation time, and energy of two singularities at M=0,1,2,3 under different viscosity coefficient ν. $t_{\max}$ is the time where the kinetic energy (KE) reaches its maximum.

	ν	0.001	0.01	0.1	1.0	
*M*						
		Yes	Yes	Yes	Yes	Stable
0		0.233	0.231	0.225	0.262	$t_{\max}$
		2.2291	2.2578	1.2707	0.1317	KE
		Yes	Yes	Yes	Yes	Stable
1		0.412	0.411	0.417	0.461	$t_{max}$
		1.2721	1.2251	0.5878	0.0588	KE
		Yes	Yes	Yes	Yes	Stable
2		0.543	0.543	0.554	0.600	$t_{max}$
		0.9513	0.8981	0.4174	0.0398	KE
		Yes	Yes	Yes	Yes	Stable
3		0.672	0.675	0.690	0.735	$t_{max}$
		0.7409	0.7056	0.3237	0.0292	KE

**Table 2:** The stability, annihilation time, and energy of two singularities at M=0,1,2,3 under different geometrical parameter β. $t_{\max}$ is the time where the kinetic energy (KE) reaches its maximum.

	β	0	−0.5	−1	
*M*					
		Yes	Yes	Yes	Stable
0		0.297	0.303	0.262	$t_{\max}$
		0.4925	0.1688	0.1317	KE
		Yes	Yes	Yes	Stable
1		0.496	0.501	0.461	$t_{max}$
		0.2830	0.0972	0.0588	KE
		Yes	Yes	Yes	Stable
2		0.634	0.639	0.600	$t_{max}$
		0.2123	0.0733	0.0398	KE
		Yes	Yes	Yes	Stable
3		0.769	0.774	0.735	$t_{max}$
		0.1693	0.0581	0.0292	KE

**Table 3:** The stability, annihilation time, and energy of two singularities at M=0,1,2,3 under different penalization parameter ε. $t_{\max}$ is the time where the kinetic energy (KE) reaches its maximum.

	ε	0.1	0.05	0.01	0.001	
*M*						
		Yes	Yes	No	No	Stable
0		0.185	0.262	−−	−−	$t_{\max}$
		0.0602	0.1317	−−	−−	KE
		Yes	Yes	Yes	Yes	Stable
1		0.215	0.461	0.006 (No annihilation)	0.005 (No annihilation)	$t_{max}$
		0.0461	0.0588	0.0106	0.0128	KE
		Yes	Yes	Yes	Yes	Stable
2		0.236	0.600	0.009 (No annihilation)	0.007 (No annihilation)	$t_{max}$
		0.0391	0.0398	0.0058	0.0065	KE
		Yes	Yes	Yes	Yes	Stable
3		0.256	0.735	0.011 (No annihilation)	0.009 (No annihilation)	$t_{max}$
		0.0338	0.0292	0.0041	0.0045	KE

**Table 4:** Time convergence rates for the director, velocity, and pressure.

Δt	$d-L^{2}$	$d-H^{1}$	$u-L^{2}$	$u-H^{1}$	$p-L^{2}$	$p-H^{1}$
$10^{-3}$	−−	−−	−−	−−	−−	−−
$5\times 10^{-4}$	0.5909	0.7283	0.9144	0.8829	0.6967	0.8225
$2.5\times 10^{-4}$	0.7568	0.8183	1.0692	1.0160	0.8641	0.9055
$1.25\times 10^{-4}$	0.8676	0.8934	1.0949	1.0530	0.9255	0.9068
$6.25\times 10^{-5}$	0.9311	0.9424	1.0669	1.0382	0.9580	0.8849

**Table 5:** Space convergence rates for the director, velocity, and pressure.

*h*	$d-L^{2}$	$d-H^{1}$	$u-L^{2}$	$u-H^{1}$	$p-L^{2}$	$p-H^{1}$
$\frac{1}{16}$	−−	−−	−−	−−	−−	−−
$\frac{1}{32}$	2.0609	1.1252	2.0821	1.1781	2.0856	1.1528
$\frac{1}{64}$	1.9523	1.1330	1.9598	1.1832	1.9829	1.1714
$\frac{1}{128}$	2.0024	0.8941	1.9800	0.8912	1.9968	0.8992
$\frac{1}{256}$	2.0277	1.0411	2.0701	1.0499	2.0311	1.0433

## Data Availability

Not applicable.
